# What do we need to know? Data sources to support evidence-based decisions using health technology assessment in Ghana

**DOI:** 10.1186/s12961-020-00550-8

**Published:** 2020-04-28

**Authors:** Samantha A. Hollingworth, Laura Downey, Francis J. Ruiz, Emmanuel Odame, Lydia Dsane-Selby, Martha Gyansa-Lutterodt, Justice Nonvignon, Kalipso Chalkidou

**Affiliations:** 1 School of Pharmacy, University of Queensland, 20 Cornwall St, Woolloongabba, QLD 4102 Australia; 2 School of Public Health, Imperial College London, London, United Kingdom; 3 iDSI, Imperial College London, London, United Kingdom; 4 Policy Planning, Monitoring and Evaluation, Ministry of Health, Accra, Ghana; 5 National Health Insurance Authority, Accra, Ghana; 6 Ministry of Health, Accra, Ghana; 7 School of Public Health, University of Ghana, Accra, Ghana; 8 Center for Global Development, London, United Kingdom

**Keywords:** Health technology assessment, Ghana, data, health information systems

## Abstract

**Background:**

Evidence-based decision-making for prioritising health is assisted by health technology assessment (HTA) to integrate data on effectiveness, costs and equity to support transparent decisions. Ghana is moving towards universal health coverage, facilitated mainly by the National Health Insurance Scheme (NHIS) established in 2003. The Government of Ghana is committed to institutionalising HTA for priority-setting. We aimed to identify and describe the sources of accessible data to support HTA in Ghana.

**Methods:**

We identified and described data sources encompassing six main domains using an existing framework. The domains were epidemiology, clinical efficacy, costs, health service use and consumption, quality of life, and equity. We used existing knowledge, views of stakeholders, and searches of the literature and internet.

**Results:**

The data sources for each of the six domains vary in extent and quality. Ghana has several large data sources to support HTA (e.g. Demographic Health Surveys) that have rigorous quality assurance processes. Few accessible data sources were available for costs and resource utilisation. The NHIS is a potentially rich source of data on resource use and costs but there are some limits on access. There are some data on equity but data on quality of life are limited.

**Conclusions:**

A small number of quality data sources are available in Ghana but there are some gaps with respect to HTA based on greater use of local and contextualised information. Although more data are becoming available for monitoring, challenges remain in terms of their usefulness for HTA, and some information may not be available in disaggregated form to enable specific analyses. We support recent initiatives for the routine collection of comprehensive and reliable data that is easily accessible for HTA users. A commitment to HTA will require concerted efforts to leverage existing data sources, for example, from the NHIS, and develop and maintain new data (e.g. local health utility estimates). It will be critical that an overarching strategic and mandatory approach to the collection and use of health information is developed for Ghana in parallel to, and informed by, the development of HTA approaches to support resource allocation decisions. The key to HTA is to use the best available data while being open about its limitations and the impact on uncertainty.

## Background

Every health system is challenged when balancing a finite budget with increasing demands on health resources. Setting effective priorities that maximise health gains necessarily requires difficult trade-offs to be made. These challenges are particularly acute in low- and middle-income countries (LMICs). Irrespective of jurisdiction, understanding trade-offs is arguably necessary to help ensure value for money and that waste is minimised [1, 2]. When setting health priorities, decisions can be better informed through the use of credible and fit-for-purpose evidence on where and how money is being spent in the health system, outcomes of interaction with the health service, and the general ‘healthiness’ of the population. Such information can be synthesised in order to understand value-for-money of current and future investments in the health system and the impact on health through increasing efficiency. Health technology assessment (HTA) represents a commonly used approach to synthesising evidence on the effectiveness and costs of an intervention, which also aims to consider social value judgements as part of a process to inform efficient and equitable resource allocation [1, 2]. While the specific methods and processes vary according to context, HTA informs health spending decisions in almost all high-income countries [3], and increasingly in middle-income countries such as Thailand [4, 5] and South Africa [6, 7], and regions including the Middle East and North Africa [8, 9]. The World Health Assembly Resolution of 2014 on Health Intervention and Technology Assessment recognizes the importance of this approach as a key element of any strategy for achieving universal health coverage (UHC) [2, 10].

The Government of Ghana has enacted legislation and earmarked funds for UHC. The National Health Insurance Scheme (NHIS), established in 2003 [11], offers a comprehensive healthcare package to all residents of Ghana but coverage is only for 38% of the population [12]. Almost half (46%) of NHIS expenditure is spent on medicines [13], where the two largest medicine classes – antibiotics (29%) and antihypertensives (14%) – account for most of the medicines expenditure [13]. In this context, an HTA pilot study was initiated by the Ghanaian Ministry of Health (MoH) in 2016 to examine the cost-effectiveness of antihypertensives in the NHIS [14]. This pilot catalysed a partnership between government and academia, demonstrating the importance of institutional collaboration in evidence-informed processes, and how HTA can inform NHIS spending decisions to improve allocative efficiency [15]. In the national medicines policy [16], the Government has outlined its vision to institutionalise HTA as part of its plan towards achieving UHC and has explicitly committed to using HTA evidence to inform prioritisation for the essential medicines list [17] and standard treatment guidelines [18].

Evidence-based policy-making depends on the availability of reliable, locally generated evidence. High-quality and timely health data creates a strong foundation for a high-functioning health system [3], but there is a marked absence of reliable health information systems in LMICs. In an important first step towards recognising and addressing shortcomings in its health data ecosystem, the Government held a forum in 2017, the Data for Sustainable Development roadmap, and committed to increasing the emphasis on enhancing routine administrative data and integrating new types of data into official statistics [2, 19, 20]. Although there is not a comprehensive suite of robust local evidence, Ghanaian policy-makers can continue to engage in evidence-informed policy-making noting the challenges for the effective implementation of HTA [2, 20]. Taking stock of the information available within the Ghanaian health system is an essential first step towards planning for the routine generation and use of HTA into the evidence-based priority-setting process in Ghana. We aimed to describe the publicly available data sources in Ghana that can support HTA and evidence-based priority-setting.

## Methods

We identified the information required to support HTA based on previous work from our group [21]. We focused on six ‘data’ domains, namely epidemiology, clinical efficacy, costs, health service use, quality of life and equity [21]. We identified data sources based on our local experience, using the internet to search the literature, Ghana government and other websites (e.g. non-government organisations), and liaising with key informants for additional input.

## Results

These six data source domains encompass many of the key elements needed for a well-conducted economic evaluation, with context-relevant details specified ex ante based on a suitable reference case [21]. iDSI developed a reference case for adaptation to local situations [22] and it provides a systematic way to inform the conduct and reporting of economic evaluations. We have summarised the main data sources available in Ghana (Table 1) and those used for the Ghana hypertension economic evaluation (Table 2) [14].

**Table 1:** Summary of key data sources for HTA in Ghana

HTA-related information	Data Source	Institution	Collection method	Equity aspect	Website	Comment
1. **Epidemiology**						
Population profile	Census	Ghana Statistical Service	Survey	Yes	http://www.statsghana.gov.gh/	Last census 2010
Demographics	Vital statistics	Births and deaths registry	Register, verbal autopsy	Unknown	http://www.eservices.gov.gh/bdr/SitePages/bdr-home.aspx	Many deaths not recorded, causes unknown
Burden of Disease	Burden of disease	Institute for Health Metrics and Evaluation	Health information system	Yes	http://www.healthdata.org/ghana	Disaggregated data – need to apply for access
DHS	DHS	DHS Programme	Survey	Yes	https://dhsprogram.com/Publications/Publication-Search.cfm?ctry_id=14&country=Ghana https://dhsprogram.com/what-we-do/survey/survey-display-437.cfm	Latest DHS was in 2014 Current DHS is being validated
MICS	MICS	UNICEF, Ghana Statistical Service	Unknown	Unknown	http://mics.unicef.org/surveys	2011 available MICS6 (2017–18) is being analysed
Disease profiles	DHIMS	GHS (CHIMG)	HIS	Yes	https://www.facebook.com/CHIMGH/?fref=ts	Access – permission required
Disease surveillance	GHS	Disease surveillance Dept., Public Health	HIS	Unknown	http://www.ghanahealthservice.org/division-scat.php?ghsdid=10&ghsscid=58	Communicable and non-communicable diseases
Ghana HIS indicators	GHS	Measure	Collation	No	https://www.measureevaluation.org/his-strengthening-resource-center/country-profiles/ghana	Some reports published Raw data may be available
2. **Clinical efficacy**						
Efficacy – trials	Trials of interventions	Industry mostly	Trials	Often sparse (Chiu – Sam)	Published literature (e.g. PubMed)	Few trials in Ghana
	Clinical Trials Department	Food & Drugs Authority	Register	No	https://fdaghana.gov.gh/index.php/clinical-trials-department/	Authorisation and monitoring of clinical trials
	Pan African Clinical Trials Registry	PACTR	Register	No	http://www.pactr.org/ International Clinical Trials Registry Platform http://www.who.int/ictrp/en/	Regional register of clinical trials conducted in Africa
Efficacy – systematic reviews	Cochrane Library	Cochrane Library, Wiley	–	–	http://www.cochranelibrary.com/help/access-options-for-cochrane-library.html	Access is provided by internet protocol country recognition
Safety	Pharmacovigilance	Food & Drugs Authority	Unknown	Unknown	https://fdaghana.gov.gh/index.php/safety-monitoring-department/	May have adverse event reporting system
Medical research	Division of Research & Development	GHS	–	No	http://www.ghanahealthservice.org/ghs-division.php?ghs&ghsdid=11	–
3. **Costs**						
Health expenditure	National Heath Accounts	MoH WHO	NHA	Unknown	https://knoema.com/WHONHA2018Feb/national-health-accounts?country=1000200-ghana	Not easily accessible
Medicines prices	NHIS list	NHIA	Central decisions	No	http://www.nhis.gov.gh/News/what-you-need-to-know-about-nhis-medicines-list-4130	Some delays in updating costs each year
	Survey	WHO & HAI	Survey	May have data for regions	http://apps.who.int/medicinedocs/en/d/Js18074en/	2004 published, 2018 survey completed (MoH)
Health services	NHIS tariffs	NHIA & GHS	Central decisions	No	Benefits package http://www.nhis.gov.gh/benefits.aspx	Not current for tariffs, cost manual is underway, based on the JLN model
	Private health insurance	PHI bodies	Claims	Possible	http://www.nhis.gov.gh/phis.aspx	–
OOP costs	DHS	–	Survey	Yes	See 2014 report	Unsure if can access raw data
4. **Service use**						
Health services	NHIS	NHIA	Claims	Yes	Benefits package http://www.nhis.gov.gh/benefits.aspx	Mostly paper-based; 30% electronic
	DHIMS	GHS (CHIMG)	HIS	Possible	https://www.facebook.com/CHIMGH/?fref=ts Centre for Health information Management Ghana	Access – permission required
	Annual report	GHS	Report	No	http://www.ghanahealthservice.org/downloads/GHS_ANNUAL_REPORT_2016_n.pdf	Published: Annual report 2016; Facts and Figures 2018
	PHI	PHI companies	Claims	Possible	http://www.nhis.gov.gh/phis.aspx	–
Healthcare Access and Quality Index	GBD study 1990–2015	Institute for Health Metrics and Evaluation	Collation	Possible	http://www.healthdata.org/research-article/healthcare-access-and-quality-index-based-mortality-causes-amenable-personal-health	Based on mortality from causes amenable to personal healthcare
5. **Quality of life**						
DALY	GBD study	Institute for Health Metrics and Evaluation	Database	NA	http://www.healthdata.org/ghana	No local disability weights
6. **Equity**						
Epidemiology	DHS	GHS	Survey	Area, gender, income, literacy	https://dhsprogram.com/what-we-do/survey/survey-display-437.cfm	Unsure if access to disaggregated data
Service use	NHIS	NHIA	Database	Districts, gender	Benefits package http://www.nhis.gov.gh/benefits.aspx	Unsure if access to disaggregated data
	DHIMS	GHS	Database	Districts	GHS access only	Unsure if access to disaggregated data
Equitable strategies	EQUIST tool	UNICEF	Collation	Districts	http://www.equist.info/en/dashboard	Considering adoption, recent study tour in India
Healthcare Access and Quality Index	GBD study 1990–2015	Institute for Health Metrics and Evaluation	Collation	Yes	http://www.healthdata.org/research-article/healthcare-access-and-quality-index-based-mortality-causes-amenable-personal-health	Based on mortality from causes amenable to personal healthcare

*CHIMG* Centre for Health information Management Ghana, *DALY* disability-adjusted life year, *DHIMS* District Health Information Management System, *DHS* Demographic Health Survey, *EQUIST* Equitable Strategies to Save Lives, *GBD* Global Burden of Disease, *GHS* Ghana Health Service, *HAI* Health Action International, *HIS* Health Insurance Scheme, *JLN* Joint Learning Network, *MICS* Multiple Indicator Cluster Surveys, *MoH* Ministry of Health, *NA* not available, *NHA* National Health Accounts, *NHIA* National Health Insurance Authority, *NHIS* National Health Insurance Scheme, *OOP* out-of-pocket, *PHI* private health insurance

**Table 2:** Ghana Health technology Assessment (HTA) hypertension case study and data sources

Data domain & Aspect	HTA model – source	Comment, gap
**1. Epidemiology**		
Prevalence of hypertension, mortality estimates, probabilities of incidence of CVD, diabetes, incidence of related complications	- Prevalence of hypertension from Ghana Statistical Service & DHS Census Data (2012, 2014)	- 2014 Ghana DHS included measurement of blood pressure for a representative sample of the population
	- Mortality rates from WHO estimated life table for Ghana	- Preferable to use estimates of incidence of adverse events from Ghana but not able to identify good quality local cohort studies with longitudinal follow-up
	- Annual probabilities of incidence of CVD + diabetes from international literature	
	- Baseline estimates of the incidence of CVD from multivariate analysis of primary care data, black African patients in the United Kingdom (QRisk2 algorithm)	
**2 Clinical efficacy**		
Effectiveness of AHM; incidence of adverse events	NICE model	- Good evidence that the effects of the main classes of antihypertensive drugs vary by ethnicity
	Meta-analyses from international literature for black African population (not specifically Ghanaian)	
		- Estimates of adverse events were not available from any West population cohort
**3 Cost**		
Antihypertensive medicines, interactions with the health system	- Unit costs of service from NHIS price for drugs on the essential medicines list, NHIS tariffs	- NHIS tariffs are updated annually but may not be publicly available
	- Daily dose and healthcare intervention assumptions from recommendations in Ghana Standard Treatment Guidelines for hypertension	- Some data not available by brand or formulation
		- Costs to other healthcare insurers, OOPE and the value of lost production due to ill-health or use of healthcare were not included in the model
**4 Service use**		
Health services and events, treatment of adverse events	- NHIS protocols	- NHIS data is restricted access
	- Expert opinion	- 30% data are available electronically
**5 Quality of life**		
DALYs, disability weights	2004 GBD study	- Ghana burden of disease study to validate GBD data
		- More recent summary estimates were not available for the conditions of interest (stroke, CHD, diabetes and heart failure)
**6 Equity**		
Impact of geography, gender, socioeconomic status, or other differentiating factors on health outcomes	Not considered in the pilot	Results could be disaggregated by geography; urban/rural using DHS data in future analyses

*AHM* antihypertensive medication, *CHD* congenital heart defect, *CVD* cardiovascular disease, *DALY* disability-adjusted life year, *DHS* Demographic Health Survey, *GBD* Global Burden of Disease, *NHIS* National Health Insurance Scheme, *OOPE* out-of-pocket expenses

### Epidemiology

This domain includes demographics, vital statistics and burden of disease. We are mindful of the quality and recognise the incentives in operations concerning data collection and analysis even for apparently well-run endeavours such as the Demographic Health Survey (DHS) and the like [23, 24].

#### Births and deaths

The Ghana Statistical Service (GSS) administers the Civil Registration and Vital Statistics system in Ghana [25]. The Civil Registration and Vital Statistics system is a focus of the Bloomberg Philanthropies Data for Health Initiative (https://mspgh.unimelb.edu.au/dataforhealth), which aims to support country systems for counting births, deaths and causes of death in order to better inform health policy; Ghana is a participating country. The GSS can provide the population profile of the country via the census (most recent was in 2010) [26].

#### Disease burden

The DHS surveys are conducted every 5 years and are available from 1988; the latest DHS was conducted in 2014 and published in 2015 [27]. The DHS is financially supported by both the Government of Ghana and USAID. The reports and data are freely available but access to more detailed (raw) data can be requested from the GSS. The GSS has been conducting the Ghana Living Standards Survey (GLSS) since 1987/8 (with the latest round being in 2017) with the aim of measuring the living conditions and well-being of the population [28]. The WHO Study on Ageing and Adult Health (SAGE) has disease burden data for Ghana for people aged 50 and above across four waves between 2002 and 2018 [29].

Information on the prevalence and incidence of a disease or condition is necessary when developing economic evaluations and related budget impact analyses, i.e. the latter forecasts expenditure, and possibly savings, usually over a period of 3–5 years. The prevalence data for any common condition (such as hypertension) is likely to be relatively straightforward to obtain using cross-sectional studies in the published literature. However, sourcing data on the incidence of disease (i.e. the occurrence of new cases in the population) is often more challenging. Incidence data are generally obtained from population-based cohort studies where participants are followed over long periods of time. These studies are necessarily expensive to establish and maintain. One potential source of incidence data is from the Ghana Health and Demographic Surveillance Systems (HDSS) that are part of the global International Network for the Demographic Evaluation of Populations and Their Health in Developing Countries (INDEPTH, www.indepth-network.org). The Ghana HDSS centres are a collaboration between the MoH, Ghana Health Service and Ghana Statistical Service. There are three surveillance centres in Ghana – Kintampo (Bono East region), Dodowa (Greater Accra region) and Navrongo (Upper East region). These centres have been following a defined geographical population for many years, so there is large scope for the centres to provide valuable data on the incidence on disease, especially non-communicable diseases (NCD), which are lacking in Ghana. The HDSS sites conduct surveys of individuals and, more recently, have linked these survey data to the district health information management system (DHMIS). Research partners, often from the United Kingdom or countries in Europe, regularly fund specific projects using the HDSS sites. There are clear policies and protocols for data access [30].

Ghana has a number of national control programmes such as those for HIV, malaria and tuberculosis [31]. These programmes calculate incidence rates by using epidemiological modelling using a variety of inputs, including reported new cases. The HIV programme also conducts sentinel surveys. The raw data for facilities is contained in the DHIMS overseen by the Centre for Health Information Management (CHIM; https://www.chimgh.org/) – MoH and Ghana Health Service. The situation of children and women are captured through the Multiple Indicator Cluster Surveys (MICS) from UNICEF and GSS; the MICS6 (2017–18) is currently being analysed but the 2011 data are available [32].

The Institute of Health Metrics and Evaluation (IHME) oversees the Disease Control Priorities Network, which strives to generate reliable, timely and comparable data on the costs and consequences of a broad range of policy options (http://www.healthdata.org/). Ghana is one of eight Disease Control Priorities Network countries, and one of four in sub-Saharan Africa (SSA). The IHME has established the Global Heath Exchange (GHDx) with a comprehensive catalogue of surveys, censuses, vital statistics and other health-related data [33]. Ghana conducted a Burden of Disease study and validated the IHME estimates based on primary data sets and stakeholder consultations with country experts on the various IHME estimates. Some discrepancies were revealed, which were subsequently updated and referred to IHME. These revised burden of disease results were vital to updates of the National Health Policy [34], the Health Sector Medium Term Development Plan [35], and the UHC roadmap for Ghana, which is still being developed (expected December 2019) [36].

### Clinical efficacy

#### Clinical trials

The major sources for clinical efficacy estimates are usually available from randomised controlled trials as published in peer-reviewed health journals and trial repositories (e.g. clinicaltrials.gov). Such trials are often conducted in developed countries with a predominantly white population, raising questions concerning the generalisability of any findings to different ethnic groups. For example, a systematic review showed that hypertensive patients of African ancestry respond better to calcium blockers and diuretics than to ACE inhibitors and β-adrenergic blockers [37]. Clinical trials in NCDs such as cardiovascular diseases and mental health disorders have been virtually non-existent in Ghana [38]. Complex clinical trials of global importance have been conducted in Ghana (e.g. bed nets, rotavirus vaccine, zinc supplements, etc.) and continue to be implemented, mainly by academic and research institutions. The challenge is that there is no central repository to access data from these trials. Evidence from clinical trials, particularly those with more complex interventions in high-income country settings, needs to be considered within the context of implementing such interventions within the Ghanaian health system. Some potential sources of more relevant clinical trials can be found at the clinical trials department of the Ghana Food and Drugs Authority (fdaghana.gov.gh). The clinical trials department authorises (by legal instrument) and monitors clinical trials in Ghana. The Pan African Clinical Trials Registry is a regional register of trials (pactr.org). The Ghana Food and Drugs Authority can also provide information on pharmacovigilance and the safety of medicines.

The recent HTA case study in Ghana for hypertension adapted the United Kingdom National Institute for Health and Care Excellence (NICE) hypertension model [14]. This work aligns with the World Health Assembly resolution of knowledge brokering and use of best practice [39]. Some are currently exploring the issue of adapting existing trials evidence and economic evaluations to suit LMIC settings [40, 41].

#### Systematic reviews and Cochrane Library

Systematic reviews, especially those from the Cochrane Collaboration, are considered the best available evidence for clinical efficacy. The Cochrane publishers (Wiley) provide free access to the Cochrane Library in over 100 countries, including Ghana (www.cochranelibrary.com/). Access is provided by internet protocol recognition removing the requirement for individual login information.

### Costs

Data on the unit costs of health services, interventions and medicines are crucial for economic evaluations and HTA [22]. When HTA has been used to inform decision-making in countries around the world, the costs used in any economic evaluation commonly tend to focus on resources available to a third-party payer. In Ghana, this would reflect the perspective of the National Health Insurance Authority. In our economic evaluation of antihypertensive medicines, the costs for medicines on the essential medicines list [17] and medical services were derived from the NHIS tariff (available on request). The NHIS tariffs are sometimes dated and a cost manual for services is being developed based on the Joint Learning Network toolkit [42]. The medicines tariffs are revised each year and are derived through a combination of surveys, manufacturer’s reference pricing, mark-ups and other considerations. It is a joint process involving the MoH, the Ghana National Drugs Programme, and provider groups. The WHO and Health Action International, in partnership with the MoH, coordinate occasional surveys with a focus on medicines’ accessibility and affordability [43]. The most recent survey was published in 2004 [44] but a survey was conducted with MoH and WHO collaboration in 2018.

In some cases, researchers may be interested in the perspective of the consumer, especially given that only four in ten people are covered by NHIS [12]. Other consumers, particularly if they are not covered by any private health insurance, may be liable for extensive out-of-pocket (OOP) payments [45–47]. The Demographic and Health Survey provides some information on OOP spending [27]. OOP payments are generally estimated using the National Health Accounts [48], the WHO Global Health Expenditures Database [49] and Government budgets. The GLSS also includes some relevant items [28]. The HDSS centre in Navrongo, together with two centres in Burkina Faso and Vietnam, aims to estimate OOP expenditure in a household survey and develop a set of questions to facilitate OOP disease-specific measurement [50]. The MoH collects information on health expenditure using the National Health Accounts from the World Bank and OECD and plans to supplement this with local data sources in 2019. They will liaise with the Finance Directorates of all agencies under the MoH and likely upload it to the upcoming National Health Observatory [51].

### Health services use

Information on the consumption of health services is vital for economic evaluations. For example, when considering an intervention to reduce a cardiovascular event such as a myocardial infarction, it is important to understand the usual treatment pathway for someone who experiences a myocardial infarction and the resulting resource use and costs. If the intervention also reduces the occurrence of stroke, then it is necessary to account for the pathways and resource implications of someone having a stroke. The main source of routinely collected health data is from largely public (and some private) service providers such as NHIS claims and the DHIMS 2. NHIS claims are recorded – the current proportion of electronic claims is 25% (4th quarter 2019; personal communication, Dsane-Selby) – the rest are paper based [52]. There are annual NHIS reports but there is some delay in making these available online [12]. The NHIS claims data would be an obvious source to explore patterns of use of health services not only at the aggregated level but also, ideally, at the individual level. The DHIMS 2 also has a vast amount of data on health services use [53].

Information on pathways of care, particularly for NCDs, is sparse. There are normative standard treatment guidelines [18] but, in the absence of any monitoring data, it is not known to what extent these are implemented in actual practice. Ghana has an essential medicines list [17, 51] that guides the medicines available for subsidy on the NHIS [54]. The MoH is working with the private sector to integrate their service data into the DHIMS. The private health insurance industry in Ghana has 12 providers and mainly covers corporate entities and less-vulnerable populations [55]. There are claims data held by the private health insurance providers, some of which has been made available for research (personal communication, Hollingworth). These data can be used to examine patterns of health service use and costs.

### Quality of life

Health outcomes are often expressed in natural units such as lives saved or a change in some clinical measure such measurement of blood pressure. Economic evaluations that only consider such measures are limited in their usefulness – the relative value of the intervention can only be ascertained within the scope of that health outcome [22]. A more useful measure is one that includes both mortality and morbidity aspects such as disability-adjusted life years (DALY) or quality-adjusted life years (QALY). Each measure requires a valuation of health states (or disability) where one is perfect health and zero is death. Disability-adjusted life years are widely used, especially in LMIC settings, and are based on the Global Burden of Disease (GBD) study linked to years of living with a disability [56].

An alternative generic measure available to researchers and decision-makers in Ghana is the QALY, often used in high-income settings but relatively uncommon in LMICs, particularly SSA [56]. Estimating QALYs relies on the use of a generic or disease-specific multi-attribute instrument to measure health-related quality of life (HRQoL). The most widely used generic measure of this type is the so-called EQ-5D (the descriptive system comprises of five dimensions) developed by the EuroQol Group [57]. Typically, the measurement of changes in HRQoL are reported directly from patients, but the utility (valuation) of these changes are based on public preferences using a choice-based method (commonly time trade-off in the case of EQ-5D). The valuation exercise is usually undertaken separately and the results are often referred to as the population tariff. However, there are no published EQ-5D (three or five level) value sets for Ghana. An orthopaedic hospital in Accra is administering a multi-attribute instrument (SF-36D) to patients before and after surgery [58]. In Ghana, some authors have evaluated HRQoL in studies encompassing university students’ wellbeing (shorter version of the WHO quality of life (WHOQOL-BREF) across four domains) [59], stroke (HRQOLISP-40 instrument across seven domains) [60], asthma (Asthma Quality of Life Questionnaire) [61], and leprosy (disease-specific) short form 20 health survey (six domains) [62]. One study (conference presentation) used the three-level version of EQ-5D in people living with HIV [63]. There is work underway to develop EQ-5D value sets within SSA; sets are now available for Zimbabwe [64] and Ethiopia [65].

### Equity

Again, there are data on equity, depending on the specific aspect and the pragmatic applicability for HTA purposes; DHS, GLSS, and the Study on Ageing and Adult Health (SAGE) have data on equity. Although not necessarily quantified (or quantifiable), HTA processes can explore context-relevant value judgements that may modify interpretation of a cost-effectiveness analysis. Equity considerations are important in priority-setting decisions. These may include aspects relating to equitable access and the provision of health services to vulnerable populations, plus issues relating to gender, socioeconomic indicators, and tribal and religious affiliations. There may also be geographical aspects to consider, including urban versus rural differences in access and outcomes. There may be a pressing need to address inequities in health outcomes for marginalised groups; to do so, data on the relevant characteristics is required.

There are some equity-relevant data available in Ghana. The sample design of the DHS provides estimates at the national and regional levels as well as for urban and rural areas. Wealth quintiles are available for households in the datasets [27]. The GLSS also provides data on wealth quintiles [28] and the census has data for the socioeconomic status at the district level [26]. Equity aspects of service use can be explored through the NHIS and the DHIMS, especially at district and facility level. The three HDSS sites can provide some data; for example, one project aimed to understand the impact of UHC on the poor and vulnerable. The HDSS at Navrongo was one of two impact of UHC study sites (the other in Vietnam) exploring health services need, access, use, quality of care and reliable measurement of socioeconomic status at the district level [66]. The GBD study collaborators published a study on the healthcare access and quality index based on mortality from causes amenable to personal healthcare in 195 countries and territories, including Ghana [67]. The EQUIST tool from UNICEF (http://www.equist.info/en/dashboard) will be linked to the DHMIS (Ghana Health Service) by 2020.

## Discussion

### Main findings

Ghana has several large data sources to support HTA (e.g. DHS, GBD and NHIS) with sufficiently rigorous quality assurance processes. There are few accessible data sources for costs and service use. The NHIS claims data are a potentially rich source of data on these but there are access limitations. The initiative to have facilities submit claims through an electronic platform presents an opportunity to harness such a rich source of data for HTA. Data on services and medicines costs are based entirely on NHIS tariffs, but these need to be regularly reviewed and updated. The 2017 value-added tax exemption on medicines should result in an average decrease of 30% in costs for medicines with the costs of selected medicines decreasing up to 70% [68]. There are almost no data for the domains of quality of life [69] and equity.

### Strengths and limitations

To the best of the authors’ knowledge, this is the first comprehensive study to describe the landscape of health system data sources to inform HTA in Ghana and could serve as a blueprint for other SSA countries [70]. The main limitation was to accurately and comprehensively identify all the sources of data for HTA [21]. We have not been able to consider aspects of quality and missing data. As such, we consider this an important starting point for a more complete description and assessment of data suitable for HTA purposes in Ghana.

WHO urges member states to develop and improve the collection of data as noted in the Health Intervention Technology Assessment Resolution [2]. Much of the data described in this paper are derived from government-sponsored information systems and census surveys, where statistics are reported to be systematically exaggerated across Africa [24, 71–73]. We did not assess the quality of the data sources in this paper but there are promising quality assurance processes to ensure data integrity for some sources [27, 30, 52]. A more comprehensive consideration of quality could be pursued in future studies.

Measures for safeguarding data should be reviewed and improved across Ghana to ensure robust, high-quality evidence capture and protect data against undue political interference [24]. The strength of any economic evaluation lies in the data that populates it, and thus improving the quality of data collected in Ghana, and indeed across Africa, will be a crucial step towards more robust evidence-informed policy decisions. We are currently reviewing the data sources used in selected published economic evaluations from SSA.

### Implications for policy and practice

In terms of epidemiological data, this important component of HTA is adequately served by data available from the GBD and DHS. However, most other contextually relevant data sources are arguably inadequate for the production of analyses that are able to rely on information most relevant to Ghanaian decision problems. Even though the epidemiological data sources are among the most reliable, there remain substantial gaps. There are several studies exploring the prevalence of NCDs, e.g. hypertension in various Ghanaian populations [74–77], but, to our knowledge, there are no studies looking at the incidence of NCDs. This would require a large-scale cohort study and it could consider the main NCDs and comorbidities of interest in Ghana, i.e. cardiovascular disease, type 2 diabetes and cancer, and the associated risk factors including hypertension and obesity. With regard to preference-based health-related quality of life measures, Ghanaian policy-makers will need to be aware of the implications of such data to inform the routine use of QALY measures in HTAs. For example, in the overwhelming majority of countries in SSA, value sets are simply not available for the most commonly, and widely understood, generic HRQoL tool, the EQ-5D (five dimensions). The adoption of QALYs is feasible in SSA [65], and awareness raising together with action and support for research efforts at developing value sets for Ghana through peer or South–South channels may be an important means of methods development [7]. This highlights the importance of having a reference case [22] that signals preference (and creates a demand) for certain types of data [78].

It is important to highlight that, for decision-making purposes, there will always be gaps in the available evidence and, when there is evidence, it may be of varying quality [79]. Analysts and decision-makers will therefore be routinely confronted by uncertainty that needs to be acknowledged and managed. Judgement will be needed in deciding whether the data used in any HTA is the ‘best available’ for the evaluation problem being examined, and the implications of any bias introduced when using sub-optimal data sources [80]. This further highlights the appropriate conceptualisation of a functioning (‘institutionalised’) HTA system as one that has both procedural and technical elements [81], is supported politically and has cooperation among key organisations [82]. In relation to the technical elements, a key foundation is establishing a framework (or reference case) for the conduct of economic evaluations in Ghana. Such frameworks not only promote consistency but serve to illuminate the key information required for decision-making purposes, and thus help drive more targeted primary research and data collection [22].

At least initially, the new HTA mechanism in Ghana may have to work with less-than-complete access to good quality, contextually relevant data and evidence, and it will likely be necessary to accept some reliance on internationally produced evidence. Adopting approaches that do not apply hierarchies of evidence in an unthinking manner [83] and emphasising ‘fitness for purpose’ may also assist in helping researchers and decision-makers in Ghana make the best use of available evidence, whether produced domestically or from international sources [84]. For example, if health utilities are required, they could be sourced from the literature and reports from HTA agencies in similar contexts [9]. Likewise, costs should ideally be from Ghanaian sources considering local care pathways but can be supplemented by data from the literature, expert clinical opinion, and costs from similar jurisdictions with appropriate adjustment. As the programme of HTA matures in both the public and private sectors, and especially if its outputs can be used to identify research gaps ideally funded by a dedicated department within the MoH as a start, and potentially a separate agency in the future, the information sources should improve in quality and constraints should lessen over time. In addition, improving existing information systems (and addressing any governance challenges) should be a priority, not least because such action will have broader health system benefits in enabling better monitoring of performance and supporting links between best practice and remuneration. Indeed, the World Health Assembly resolution on HITA [2] rightly encourages member states to strengthen the routine collection of health system data as a necessary step towards achievement of UHC. The key to HTA is to use the best available data while being open about its limitations and the impact on uncertainty.

## Conclusions

It will be critical that an overarching strategic and mandatory approach to the collection and use of health information is developed for Ghana in parallel to, and informed by, the development of HTA approaches to support resource-allocation decisions. In this context, donors have a critical role in emphasising and supporting the development of health information systems, especially in those settings transitioning from external aid. The latter is particularly important given the track record of aid-funded data collection, which has been mostly about data to ensure progress against globally set targets (Millennium Development Goals, Sustainable Development Goals) as opposed to data needed by countries in order to run their systems to meet these targets [24]. The gathering of quality data to enhance HTA assessments should also be seen as a political choice that the country should champion. This is the only way to achieve UHC in a cost-effective way.

## Data Availability

All data generated or analysed during this study are included in this published article.

## Abbreviations

DHIMS: District Health Information Management System
DHS: Demographic Health Survey
EQ-5D: EuroQol five dimensions
GBD: Global Burden of Disease
GLSS: Ghana Living Standards Survey
GSS: Ghana Statistical Service
HDSS: Health and Demographic Surveillance Systems
HRQoL: health-related quality of life
HTA: health technology assessment
IHME: Institute of Health Metrics and Evaluation
LMICs: low- and middle-income countries
MICS: Multiple Indicator Cluster Surveys
MoH: Ministry of Health
NHIS: National Health Insurance Scheme
NCD: non-communicable diseases
OOP: out-of-pocket
QALYs: quality-adjusted life years
SSA: sub-Saharan Africa
UHC: universal health coverage
